# Immunological consequences of using three different clinical/laboratory techniques of emulsifying peptide-based vaccines in incomplete Freund's adjuvant

**DOI:** 10.1186/1479-5876-4-42

**Published:** 2006-10-23

**Authors:** Yi T Koh, Sean A Higgins, Jeffrey S Weber, W Martin Kast

**Affiliations:** 1 Dept of Molecular Microbiology & Immunology, University of Southern California, 1501 San Pablo St. Los Angeles, CA 90033, USA; 2 Dept of Medicine and Norris Comprehensive Cancer Center, University of Southern California, 1501 San Pablo St. Los Angeles, CA 90033, USA

## Abstract

Incomplete Freund's adjuvant (IFA) serves as a carrier for water-in-oil emulsion (W/O) vaccines. The stability of such emulsions greatly affects vaccine safety and efficacy since continued presence of antigen depots at lymphoid organs releasing low-level antigens is known to stimulate a potent immune response and high-level systemic release of antigens can lead to tolerance. W/O emulsions for the purpose of clinical and laboratory peptide-based vaccinations have been prepared using the techniques of syringe extrusion, vortex or high-speed homogenization. There is no consensus in the field over which technique would be best to use and no immunological data are available that compare the three techniques. In this study, we compared the immune responses induced by a peptide-based vaccine prepared using vortex, syringe-extrusion and homogenization. The vaccination led to tumor rejection by mice vaccinated with the peptide-based vaccine prepared using all three techniques. The immunological data from the *in vivo *cytotoxicity assay showed a trend for lower responses and a higher variability and greater range in the immune responses induced by a vaccine that was emulsified by the vortex or homogenizer techniques as compared to the syringe-extrusion technique. There were statistically significant lower numbers of IFNγ-secreting cells induced when the mice were vaccinated with a peptide-based vaccine emulsion prepared using the vortex compared to the syringe-extrusion technique. At a suboptimal vaccine dose, the mice vaccinated with a peptide-based vaccine emulsion prepared using the vortex technique had the largest tumors compared to the syringe-extrusion or the homogenizer technique. In the setting of a busy pharmacy that prepares peptide-based vaccine emulsions for clinical studies, the vortex technique can still be used but we urge investigators to take special care in their choice of mixing vessels for the vortex technique as that can influence the stability of the emulsion. However, in instances where the optimal dose is unknown, we caution investigators against using the vortex technique to prepare the peptide-based vaccine emulsions. Overall, we report that all three techniques can be used to prepare peptide-based vaccine emulsions under optimal dose conditions and we discuss important details regarding the proper preparation of the emulsions.

## Background

Adjuvants augment and potentiate immunological responses and have been used to increase the efficacy of vaccines. Mineral oils were first used as an adjuvant in 1916 when Le Moignic and Pinoy found that a suspension of killed *Salmonella typhimurium *in vaseline oil with lanolin as an emulsifier increased immune responses[1]. Two decades later, Jules Freund developed a potent adjuvant, Freund's complete adjuvant (FCA) which was basically a mineral (paraffin) oil containing killed mycobacteria. FCA was developed in the mid-1930s following the observation that guinea pigs infected with *Mycobacterium tuberculosis *produced higher titer antibody responses following immunization than non-infected animals[2]. Freund later found that immunization of the protein antigen in a water-in-paraffin oil emulsion without killed mycobacteria was just as effective in increasing and prolonging antibody formation[3], leading to the development of incomplete Freund's adjuvant (IFA) which is a water-in-oil (W/O) emulsion without killed mycobacteria.

Although FCA is one of the most effective adjuvants known, it results in generalized granulomatous proliferation in various organs [4-6], severe pain and distress[7] and is not approved for human use. The prototypical FCA and IFA contained impurities in the crude mineral oil that led to the reports of site-of-injection toxicities [8-10]. Since then, efforts aimed at increasing the adjuvant potency of IFA while reducing toxicity have led to new generations of highly purified mineral oils such as the Montanide incomplete Seppic adjuvants (ISAs) (Seppic, Paris, France) that have been approved for both human and veterinary use[11,12]. IFA types of adjuvants such as Montanide ISA-51 and Montanide ISA-720 are now widely used in cancer immunotherapy strategies in patients[13,14] and can be combined with other adjuvants such as immunostimulatory CpG oligodeoxynucleotides[15] that trigger the innate immune response through Toll-like receptors.

An emulsion is a thermodynamically unstable mixture of two immiscible liquids. The peptide IFA W/O emulsions consist of the peptide antigen dissolved in the phosphate buffered saline (PBS) liquid phase dispersed in the mineral oil phase. A well-prepared IFA emulsion is a thick, viscous, white mixture that does not disperse when dropped into a beaker of water. This property of the emulsion allows it to maintain an antigen depot that does not disperse readily into the aqueous environment protecting the peptide antigen from rapid degradation. The integrity of emulsions is classically determined by a water-drop test where a small drop of emulsion is placed on the surface of room temperature distilled water[16]. The emulsion has to be mixed to a point where an emulsion droplet does not fall apart upon contact with the water and form an oily slick over the surface of the water. When the IFA W/O emulsion falls apart, antigen is released into the system. The kinetics of antigen release has been shown to determine the level of immune response in murine models [17-19]. Generally, the regulated slow release of antigen into the host stimulates an immunogenic response while a rapid systemic distribution of antigen can lead to the induction of T cell tolerance [17-19]. The integrity of W/O emulsions is important because the large multimolecular emulsion aggregates can promote uptake by macrophages and dendritic cells, facilitating their transport into the lymphatics where the localized small depots of antigens persist to stimulate the immune response[20,21].

This paper aims to compare the immune responses induced by a peptide-based vaccine prepared using one of the three emulsification techniques (syringe-extrusion, the vortex and the homogenizer) and to give advice to investigators in the field on the best technique to use. In the conventional syringe-extrusion technique[22], the aqueous phase containing the antigen is forced into an equal volume of the oil-phase adjuvant either through double-hubbed small-bore needles or three-way stopcocks[16,23]. The vortex method[24] is simple and has been used to prepare veterinary and human vaccines[25]. The water phase and the mineral oil phase is placed in the same tube and mixed by vortexing until an emulsion is formed[26]. Industrially, W/O emulsions are prepared using high-speed homogenization[27]. We have successfully used the homogenizer technique to prepare W/O emulsions that have been used to induce immune responses in mice[17] and we will now compare the level of immune responses induced by peptide emulsions vaccines prepared using the syringe-extrusion, the vortex and the homogenizer techniques. The conventional syringe-extrusion technique is probably the most commonly used technique, but is very tedious and laborious. Although W/O emulsions have been prepared in clinical trials by vortexing on a table-top vortex and used for cancer immunotherapy of patients[13,28], there is a debate within the field about the quality of emulsions prepared using the vortex versus syringe-extrusion techniques. The syringe-extrusion method is thought to be superior to the vortex because the emulsions obtained appear to be more viscous. The homogenizer uses high-speed shear force to emulsify the W/O emulsion, is convenient and produces an emulsion of equal or greater apparent viscosity compared to the syringe-extrusion method. The homogenizer is a more expensive piece of equipment compared to the glass syringes used in the syringe-extrusion technique or to the vortex and is therefore less commonly used compared to the other two.

## Materials and methods

The C3 cell line is a tumorigenic human papilloma virus (HPV) 16 and EJ-ras transformed C57BL/6 mouse embryo cell that expresses the E7 early protein[29]. The C3 cell line was maintained in Iscove's Modified Dulbecco's Medium (IMDM) supplemented with 10% heat-inactivated fetal calf serum, 2 mM l-glutamine (Gibco-Invitrogen, Carlsbad, CA), 50 mM 2-mercaptoethanol (EMD Chemicals, Gibbstown, NJ) and 100 μg/ml of kanamycin (Sigma-Aldrich, St. Louis, MO). Cells were incubated at 37°C, 5% CO_2_. All peptides were synthesized by the Microchemistry core facility of the Norris Comprehensive Cancer Center or provided by Dr. Steve Meredith of the University of Chicago. HPV-16 E7 peptide (RAHYNIVTF^49-57^) is a H-2D^b^-binding peptide that mediates rejection of C3 tumors in C57BL/6 mice[29]. The prostate-specific antigen PSA peptide (VTWIGAAPL ^(-3)-6^) binds to both H-2D^b ^and H-2K^b ^(unpublished results) and was used as an irrelevant peptide control for the immune assays.

### Peptide emulsion preparation

**Figure 1:**
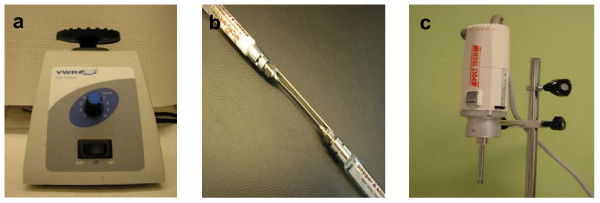
**Vortex, syringe-extrusion and homogenizer equipment used in this study**. (a) VWR Mini Vortexer, (b) Glass syringes with 22-gauge connector reinforced with a steel bar, (c) Polytron PT2100 homogenizer

100 mg/ml HPV 16 E7 peptide stocks were dissolved in dimethylsufoxide (DMSO) and stored as 1 mg aliquots at -80°C. HPV 16 E7 peptide was dissolved in PBS at 2 mg/ml and emulsified at a 1:1 ratio with incomplete Freund's adjuvant (IFA) (Rockland Immunologicals, Gilbertsville, PA) to obtain a final concentration of 1 mg/ml. To prepare the emulsion by vortexing, the PBS-IFA mixture was placed in 1.7 ml or 2 ml Eppendorf tubes, taped flat down on the vortex head (VWR, West Chester, PA) and vortexed for ten minutes at a maximum speed of 3, 200 rpm (Fig. 1a). Emulsification by syringe-extrusion was carried out by passing the PBS-IFA mixture through a reinforced 22-gauge connector between two glass syringes for ten minutes (Fig. 1b). Emulsion preparation by homogenizer was achieved using the polytron PT2100 (Kinematica AG, Littau-Lucerne, Switzerland) (Fig. 1c). The integrity of the emulsions were tested using the water-drop method and were deemed properly emulsified when they did not disperse immediately after a droplet was dropped into a beaker of water. Percentage phase separation of the emulsion is calculated as follows [30]:

% phase separation of the emulsion = height of the oil phase/(total height of emulsion sample × initial fraction of oil in emulsion) × 100

### Vaccination

Eight to ten week old C57BL/6 mice were purchased from Taconic (Hudson, NY), vaccinated subcutaneously with 100 μg of HPV16 E7 peptide emulsified in 1:1 PBS:IFA mixture and boosted two weeks later with another 100 μg of HPV16 E7 peptide emulsified in 1:1 PBS:IFA mixture. Seven days after the boost, four mice were sacrificed for immune assays. For the tumor protection studies, eight mice were challenged with 5 × 10^5 ^C3 cells ten days after the boost. In the tumor protection studies using a suboptimal dose of vaccine, 25 μg of HPV16 E7 peptide was used to vaccinate the mice subcutaneously and the mice were challenged with 10^6 ^C3 cells.

### *In vivo *cytotoxicity assay

Spleens were harvested from eight to ten week old naïve C57BL/6 mice and the splenocytes were used as target cells for an *in vivo *cytotoxicity assay as previously described[31]. The splenocytes were pooled and the red blood cells lysed using ammonium chloride/potassium (ACK) lysing buffer. The splenocytes were then washed and incubated with 0.5 μg/ml E7 peptide or PSA peptide as an irrelevant peptide control at 37°C, 5% CO_2 _for 90 min with gentle agitation every 30 min. The splenocytes were then washed three times and resuspended in 0.1%BSA in PBS. The E7 peptide-pulsed targets were labeled with 10 mM carboxyfluoroscein succinimidyl ester (CFSE) (Molecular Probes Invitrogen, Carlsbad, CA) and the PSA peptide-pulsed targets labeled with 1 mM CFSE at 37°C, 5% CO_2 _for 15 min. Ice-cold 10% IMDM was used to stop the labeling reaction and the target cells washed twice. The target cells were re-suspended at a final concentration of 50 × 10^6 ^cells/ml and mixed at a 1:1 ratio. 200 μl of target cells were injected i.v into the retro-orbital vein of vaccinated and control mice. The vaccinated and control mice were sacrificed the next day and their spleens harvested. The red blood cells were lysed by ACK lysing buffer and 5 × 10^6 ^cells used for FACS analysis by gating on the CFSE positive splenocytes. The percentage specific lysis of the E7 peptide pulsed targets were indicated by the loss of the CFSE^high ^E7-pulsed population relative to the control CFSE^low ^PSA-pulsed population. % specific lysis was calculated by the following formula :

% specific lysis = [(% irrelevant population-% relevant population)/% irrelevant population] × 100

### IFN-γ Elispot

Multiscreen-HTS IP plates (Millipore, Billerica, MA) were washed with 50 μl of 70% ethanol, rinsed twice with 200 μl of sterile water and washed again with 200 μl sterile PBS. IFN-γ capture antibody (R4-6A2) (BD Pharmingen, San Diego, CA) was coated onto the plates at 5 μg/ml overnight at 37°C. Capture antibody was discarded the next day by flicking and the plates washed with 0.5% PBS-TWEEN-20 (PBST) and then twice with 200 μl PBS. The plates were blocked with 10% IMDM for 1 hr at 37°C. Lymphocytes were plated at 2-fold serial dilutions along with 10% IMDM containing 5 μg/ml IL-2 and 1 μg/ml E7 peptide or PSA peptide as an irrelevant peptide control. After 48 hrs incubation at 37°C the cells were discarded and the plates washed five times with 0.5% PBST and five times with water. 100 μl of 5 μg/ml of biotinylated IFN-γ (XMG1.2) (BD Pharmingen) was added to the wells and incubated at 4°C overnight. Biotinylated anti-IFN-γ was discarded by flicking and the plates washed six times with 0.05% PBST. 100 μl of 1 μg/ml horseradish peroxidase-conjugated strepavidin (Sigma-Aldrich) in 0.05% PBST and 1% BSA was added to the wells and incubated for 1 hr at room temperature. The plates were washed three times with 0.05% PBST and three times with PBS. The substrate, 3-amino-9-ethylcarbazole (AEC) (Sigma-Aldrich) was dissolved in dimethylformide (DMF) (Sigma-Aldrich) and diluted in 0.1 M NaAc buffer pH 5.0. 5 μL of H_2_O_2 _was added per 10 ml of AEC substrate solution just prior to adding substrate to the wells. Substrate was allowed to develop between 15 min to 60 min. Then the plates were washed and allowed to dry. The spots were counted, averaged and subtracted from the background spots counted in the wells stimulated with the irrelevant PSA peptide. This number was converted to the average number of IFN-γ-secreting cells per 10^6 ^splenocytes present in the immunized or control mice.

### Tumor induction

C3 cells in the logarithmic growth phase were trypsinized, washed and passed through a 70 μm cell strainer (BD Falcon) to obtain single-cell suspensions. The numbers of viable cells were determined by trypan blue exclusion and re-suspended in serum-free Hank's balanced salt solution (HBSS) at the 5 × 10^6 ^cells/ml. Male C57BL/6 mice were *s.c*. injected on the right flank with 5 × 10^5 ^cells using a 25-gauge needle. Tumor growth was measured twice a week in three dimensions. Tumor volume was calculated by multiplying the length, width and height of the tumor.

### Statistical analysis

Data were graphed used the Prizm 4 software Macintosh (Graphpad Software Inc. San Diego, CA. The Student's t-test (one-tailed) was carried out using the Prizm 4 software. The Levene's test for the equality of variances was carried out using the SPSS 12.0 software for Windows (SPSS Inc. Chicago, IL).

## Results

### Physical stability of peptide emulsions

**Figure 2:**
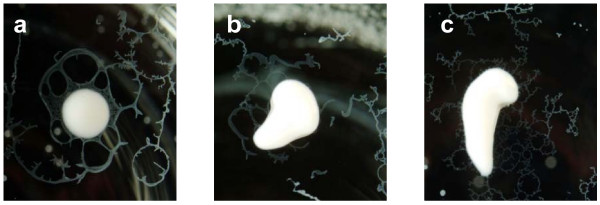
**Water-drop test for emulsion stability**. Representative photographs of emulsion droplets prepared by vortex, syringe-extrusion or homogenizer. (a) Emulsion was prepared using the vortex technique by vortexing the emulsion at 3,200 rpm for ten minutes in a 2 ml Eppendorf tube to reduce aeration. (b) Emulsion was pushed back and forth between two glass syringes connected by a 22-gauge connecter for ten minutes in the syringe-extrusion technique. (c) Emulsion was subjected to high-speed homogenization for ten minutes with an attached blade at 30,000 rpm.

In order to demonstrate the integrity of the emulsions prepared in this study, a droplet of the peptide emulsion was dropped into a beaker of distilled water. Traditionally, an emulsion is deemed properly prepared for vaccination if the droplet retains its shape on the surface of the water. The emulsions prepared using the vortex (Fig. 2a); syringe-extrusion (Fig. 2b) or the homogenizer (Fig. 2c) techniques were able to retain their integrity and did not disperse upon contact with the water. The emulsion prepared using the vortex technique appeared to be less viscous but was still able to retain its integrity in the water-drop test.

**Figure 3:**
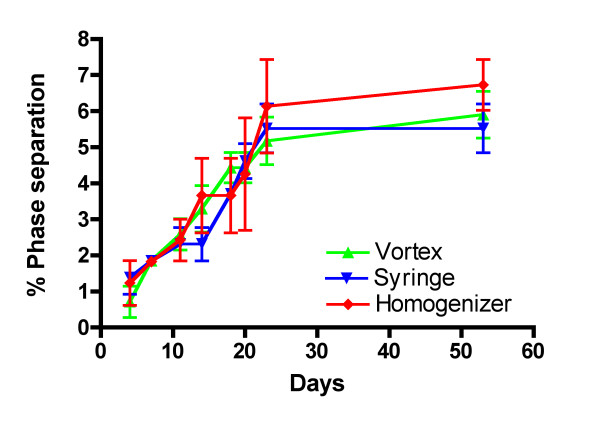
**Phase separation**. Physical stability of W/O emulsions prepared using vortex, syringe-extrusion or homogenizer for ten minutes as determined by the separation of the oil phase from the emulsion. % phase separation is measured by taking the height of the oil phase/(total height of emulsion sample × initial fraction of oil in emulsion) × 100. The mean % phase separation and standard error of the mean of four emulsion samples prepared using vortex, syringe-extrusion or homogenizer is shown over time. Figure shown is representative of three experiments.

Emulsions are not in equilibrium and the different components will separate over time. The measurement of the separation of the oil phase from the rest of the emulsion is another crude determinant of emulsion stability. The emulsions prepared using syringe-extrusion, homogenizer and vortex were aliquoted into 1 ml sample glass vials, left to sit undisturbed, and the phase separation of the oil from the emulsion measured over time. We saw no appreciable differences in the phase separation of the emulsions prepared using the different techniques (Fig. 3).

### All three techniques of preparing emulsions induce immune responses

**Figure 4:**
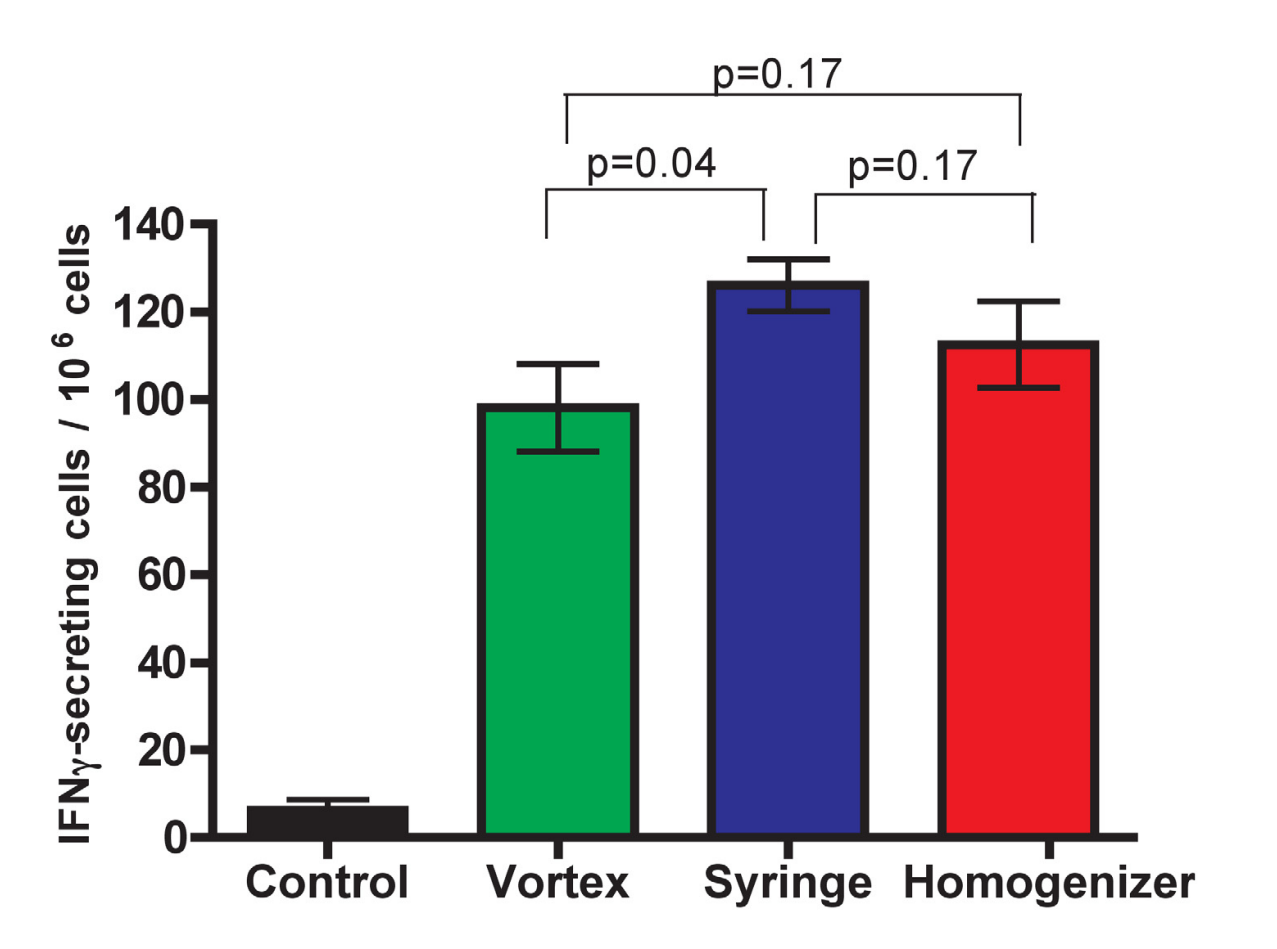
**IFN-γ ELISPOT**. C57BL/6 mice (n = 4) were immunized with E7 peptide emulsified using vortex, syringe-extrusion or homogenizer for ten minutes and boosted two weeks later. One week after the boost, the mice were sacrificed and their lymph nodes harvested. E7peptide-specific response was measured by IFN-γ secreted after subtracting background IFN-γ secretion in the presence of PSA peptide as an irrelevant control. Control mice (n = 4) were vaccinated with PBS:IFA emulsion without peptide. The mean and standard error of the mean of each group is shown below. Figure shown is representative of three experiments. A Student's t-test (one-tailed) showed that there was a significant difference between the vortex versus the syringe-extrusion technique (p = 0.04) but no statistically significant differences between the homogenizer versus the vortex technique (p = 0.17) or the homogenizer technique versus the syringe-extrusion technique (p = 0.17).

**Figure 5:**
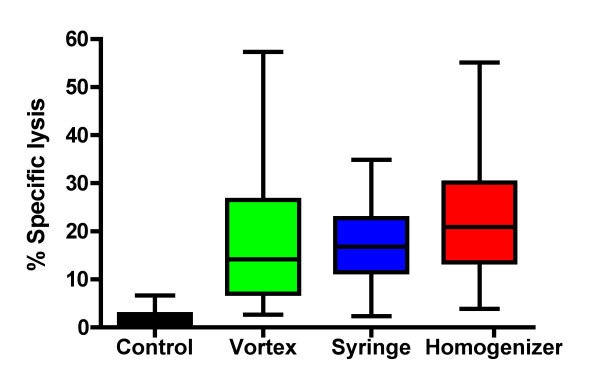
***in vivo *cytotoxicity assay**. C57BL/6 mice (n = 4) were immunized with E7 peptide emulsified using vortex, syringe-extrusion or homogenizer for ten minutes and boosted two weeks later. Control mice (n = 4) were vaccinated with PBS:IFA emulsified by syringe-extrusion for ten minutes without peptide. One week after the boost, the mice were sacrificed and their spleens harvested. Figure is a box-plot showing the five-number summary (median, maximum, minimum, 25^th ^and 75^th ^quartile) of the % specific lysis of E7 peptide-pulsed target cells after subtracting background killing of irrelevant peptide control PSA peptide-pulsed cells collected over five experiments. Median percentage specific lysis is 13% for the vortex group, 17% for the syringe-extrusion group and 21% for the homogenizer group.

Eight to ten week old C57BL/6 mice were vaccinated and boosted two weeks later with 100 μg of HPV16 E7 peptide in order to test the vaccination efficacy of the peptide/IFA emulsions prepared using the vortex, syringe-extrusion and homogenizer techniques. A week after the boost, the mice were sacrificed and their lymph nodes and splenocytes harvested for immune assays (IFNγ-Elispot assay and *in vivo *cytotoxicity assay respectively). From the IFNγ-Elispot assay shown in figure 4, there was a statistically significant difference between the numbers of IFNγ-secreting cells induced by the W/O emulsion peptide vaccine prepared using the vortex versus the syringe-extrusion techniques (p = 0.04). However there were no statistically significant differences between the homogenizer technique with the vortex (p = 0.17) or with the syringe-extrusion technique (p = 0.17). Statistical analysis of the data obtained from the *in vivo *cytotoxicity assay (Fig. 5) showed that there were no statistically significant differences in the mean percentage specific lysis of the vaccinated mice from the vortex, syringe or homogenizer groups. The variance in specific lysis values was found to be statistically significantly greater for mice vaccinated with the vortex compared to the syringe-extrusion (p = 0.03) and homogenizer (p = 0.05). Although our data do not conclusively prove that the peptide-based vaccine emulsions prepared using the vortex technique performs worse than the syringe-extrusion group or the homogenizer group, we did observe a trend that the immune responses induced were often weaker. This was reflected by the statistically lower numbers of IFNγ-secreting cells induced in the Elispot assay and the lower median percentage specific lysis in the vortex group (median = 13%) compared to the syringe-extrusion (median = 17%) or homogenizer (median = 21%) in the *in vivo *cytotoxicity assay.

### All three techniques of preparing emulsions provided protection against tumor challenge

The immunological readouts of W/O emulsion vaccines showed that there were no statistically significant differences in the mean percentage specific lysis among the three techniques in the *in vivo cytotoxicity *assay but there were fewer IFNγ-secreting cells induced by the peptide-based vaccine emulsions prepared using the vortex compared to the syringe-extrusion technique. We also observed a trend that the vortex group would often perform slightly worse and the syringe-extrusion group had a smaller variance in percentage specific lysis values than the vortex or the homogenizer group. It is difficult to ascertain what numbers of IFNγ-secreting cells or what percentage specific lysis in the immunological experiments would be enough to induce tumor protection *in vivo*. Therefore we challenged the mice with a tumorigenic dose of C3 tumor cells. A vaccination strategy administering two doses of 100 μg HPV-16 E7 peptide at a two-week interval has been shown to mediate rejection of C3 tumors in C57BL/6 mice[29].

**Figure 6:**
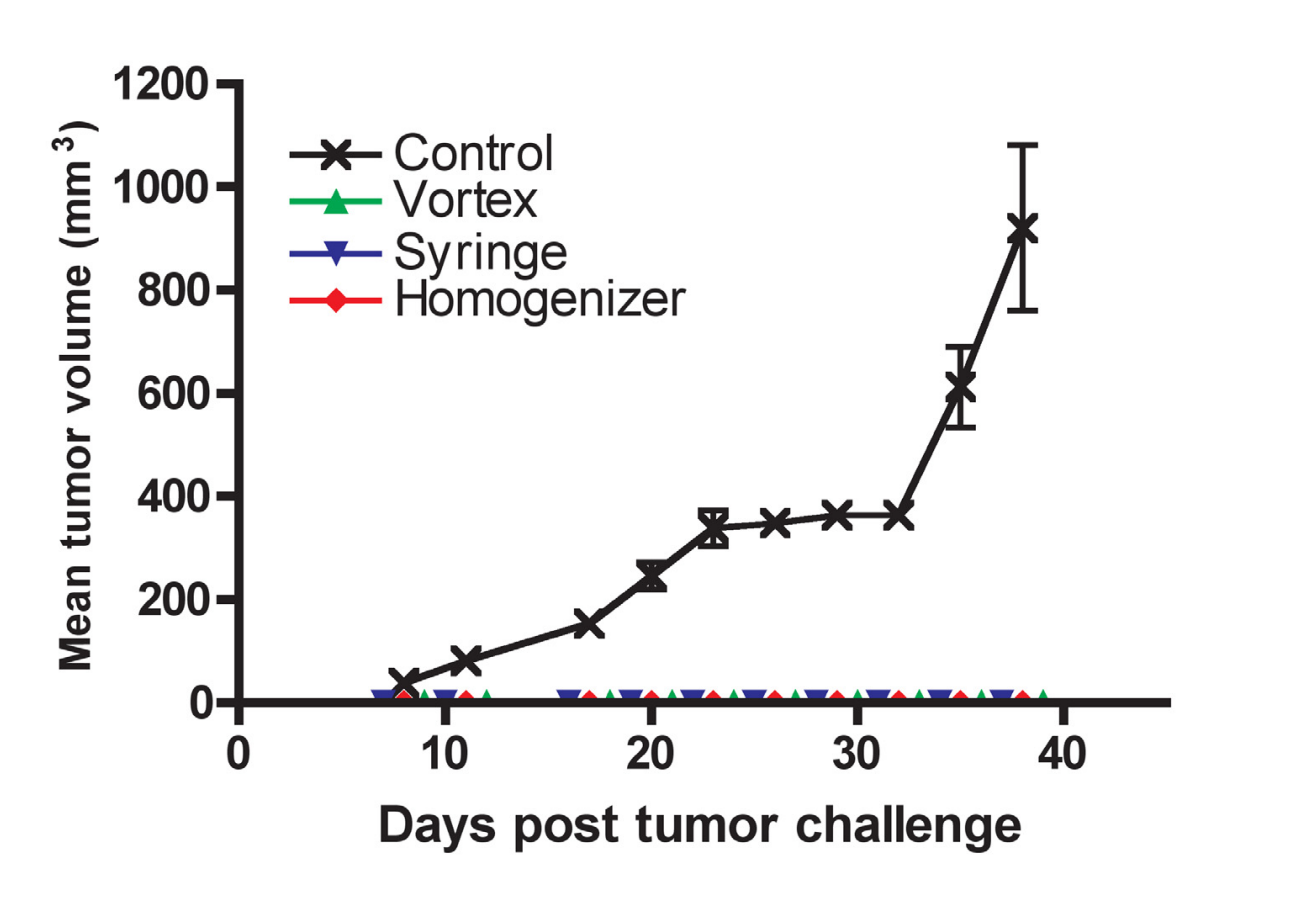
**Tumor challenge in optimal vaccine dose conditions**. C3 challenge of vaccinated and control mice (n = 8) with E7-expressing C3 cells. C57BL/6 mice (n = 8) were immunized with 100 μg E7 peptide emulsified using vortex, syringe-extrusion or homogenizer for ten minutes and boosted two weeks later. Ten days after the boost, the mice were challenged with 5 × 10^5 ^C3 cells. The control mice developed tumors but all the vaccinated mice successfully rejected the C3 cells. The mean tumor volume and standard error of the mean of each group of mice over time is shown. Figure shown is representative of four experiments.

**Figure 7:**
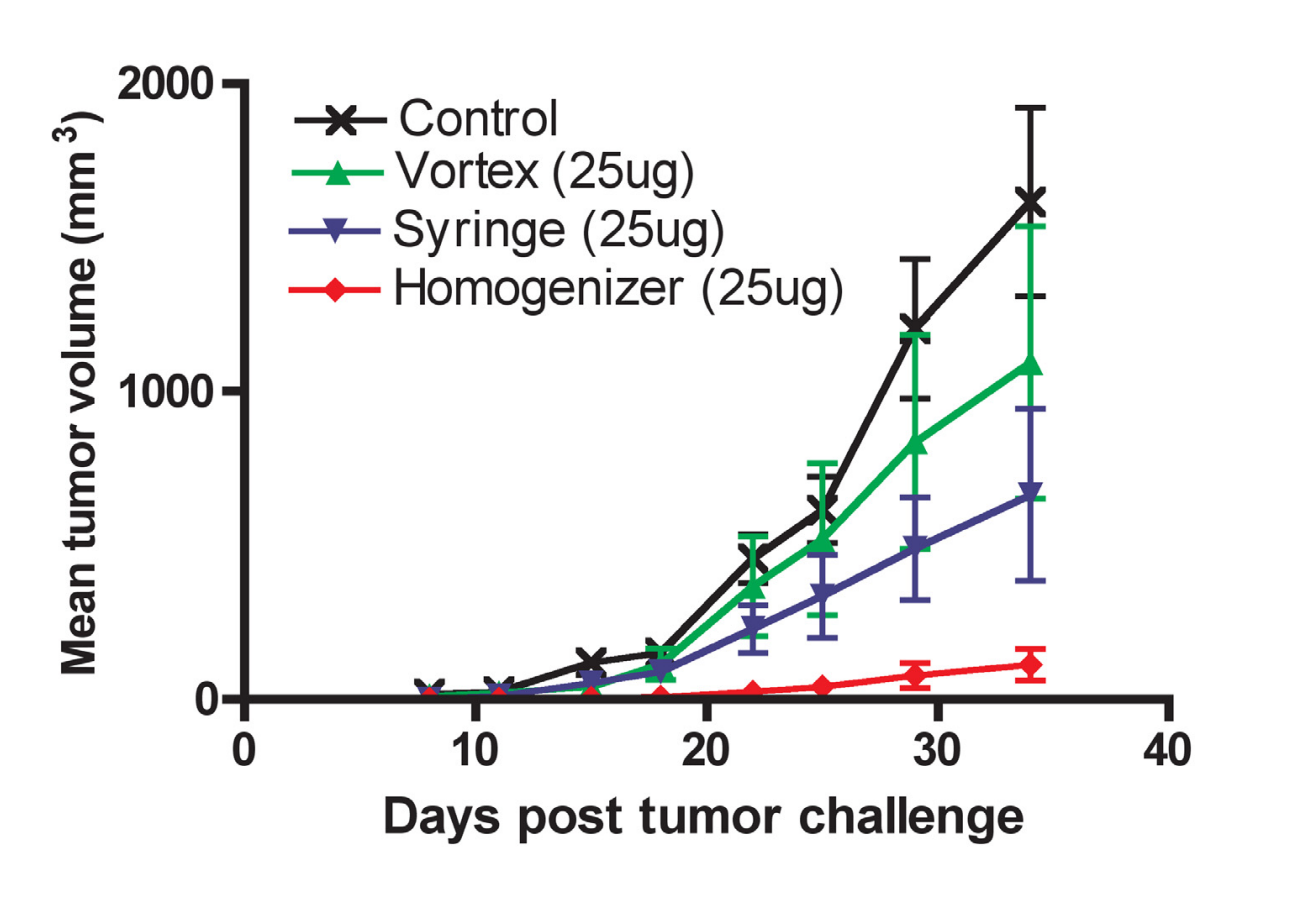
**Tumor challenge in suboptimal vaccine dose conditions**. C3 challenge of vaccinated and control mice (n = 8) with E7-expressing C3 cells. C57BL/6 mice (n = 8) were immunized with 25 μg E7 peptide emulsified using vortex, syringe-extrusion or homogenizer for ten minutes and boosted two weeks later. Ten days after the boost, the mice were challenged with 10^6 ^C3 cells. The control mice developed tumors. Four out of eight of each group of the vaccinated mice successfully rejected the C3 cells. Figure shown is the tumor growth over time of the four mice from each of the vaccinated groups that did develop tumors compared to the control mice. The mean tumor volume and standard error of the mean of each group of mice is shown. Figure shown is representative of two experiments. Using a Student's t-test (one-tailed), the vortex did not differ statistically significant from the syringe-extrusion technique (p = 0.21), but the homogenizer was statistically significantly different from the vortex (p = 0.02) and the syringe-extrusion technique (p = 0.02).

In the tumor challenge experiment shown in figure 6, all of the vaccinated mice were protected from tumor challenge. Two injections of 100 μg of HPV-16 E7 peptide protects all the vaccinated mice and this peptide dose might be at the plateau phase of the dose-response curve since Daftarian et al. have successfully used the HPV-16 E7 peptide at a dose of 50 μg [32]. We therefore reduced our vaccine dose to 25 μg of HPV-16 E7 peptide and increased the tumor challenge dose to 10^6 ^C3 cells. This resulted in half of the mice vaccinated in each group developing tumors (Fig. 7). From the data obtained, the emulsion prepared using the vortex technique provided the least protection since the mice that did develop tumors had almost the same size tumors compared to the control mice that did not receive the HPV-16 E7 peptide. The mice vaccinated with the emulsion prepared using the syringe extrusion technique had smaller but not statistically significant tumors compared to the vortex group (p = 0.21). The peptide-based vaccine emulsion prepared using the homogenizer technique was able to control the tumor growth the best.

## Discussion

The purpose of this study was to determine if there was any immunological difference in the W/O emulsions prepared using vortex, syringe-extrusion or homogenizer techniques. In order to compare the techniques, we had to ensure that stable emulsions were prepared properly using the three techniques. Many investigators report not being able to obtain a stable emulsion using the vortex although it is a method that has been used to prepare peptide emulsions in the clinic[13,24,25,28]. In our own hands, we have found that the size of the mixing vessel in which the emulsion is prepared using the vortex made a major difference in its stability as determined by the ability of the droplets to retain their shape when dropped into a beaker of water. The vortex technique can potentially introduce aeration into the emulsion system if the emulsification was not carried out in a sufficiently small mixing vessel or if there was too much splashing of the emulsion mixture during the vortex process. Aeration must be avoided during the process of emulsification because air introduces a third phase into the emulsion system that destabilizes the emulsion[33]. Even when the emulsions are prepared using the conventional syringe-extrusion technique, introducing air into the syringes would also result in an unstable emulsion. Investigators should only use glass syringes when preparing emulsions using the syringe-extrusion technique as the rubber stopper can react chemically with the mineral oils, potentially contaminating the mixture and increasing the amount of physical pressure needed to prepare the emulsions. Emulsion recovery using the syringe-extrusion is approximately 50%–75%, so volumes in excess to the amount required should be prepared when using this method[22].

The integrity of the W/O emulsions is an important consideration in vaccine efficacy because if the emulsion falls apart immediately then the peptide antigens it carries would be released and degraded rapidly, yielding an ineffective vaccine. Furthermore, it has been shown that the pharmacokinetic difference between a T-cell tolerizing peptide and a T-cell activating peptide was that the T-cell tolerizing peptide spread through the body sixteen times faster and was cleared two times quicker than the T-cell activating peptide[17]. In the case of a peptide-based vaccine emulsion, if the emulsion is unstable, it will lead to the rapid release of a high dose of peptide into the circulating system. This can lead to activation-induced cell death by the presence of an overwhelming amount of antigen and tolerization rather than activation of the immune system through presentation of the peptide on non-professional antigen-presenting cells[18]. For immunologists, the integrity of the vaccine emulsion is tested using a subjective water-drop method where the emulsification process is considered to be satisfactory if an emulsion droplet retains its integrity on the surface of a beaker of water. The emulsions prepared using the vortex technique was shown in the water-drop test to deform slightly on the surface of the water, but it was still stable enough to maintain its spherical shape and its stability seemed comparable to the other two techniques (Fig. 3) as determined by phase separation. Our data show that the vortex emulsion was just as effective in inducing an immune response that protected against tumor challenge at an optimal vaccine dose (Fig. 6). There were statistically significantly lower responses observed in the average numbers of IFNγ-secreting cells induced (Fig. 4) by the vortex versus the syringe-extrusion technique (p = 0.04), but there was no statistically significant difference in the mean percentage specific lysis against the E7 peptide-pulsed targets (Fig. 5) among the mice that were vaccinated with emulsions prepared using the vortex, syringe-extrusion or homogenizer. We also observed that there was a greater variance in the results obtained from the vortex group compared to the syringe-extrusion group (Fig. 5). The median value of the percentage specific lysis obtained from mice vaccinated with the peptide vaccine prepared by vortex was also lower compared to the syringe-extrusion or homogenizer (13% versus 17% versus 21% respectively). The greater variance in percentage specific lysis indicates that the vortex technique may not produce emulsions as uniform as the syringe-extrusion method. Decreased median values for the percentage specific lysis obtained from mice vaccinated with the peptide vaccine prepared by vortex may be the result of the non-random or incomplete distribution of the immunogen within the emulsion prepared by the vortex technique. The clinical implication of these findings is that the antigen-release profile of emulsions prepared using the vortex technique may be less consistent than the emulsions prepared using the syringe-extrusion technique. This increases the variability of the vaccine in its induction of the immune response. In some cases, it would induce a significantly higher response and in others, it results in an inferior immune response compared to the syringe-extrusion as reflected by the distribution of percentage specific lysis values in the *in vivo *cytotoxicity assay compared with the other two techniques. Our tumor challenge experiments were carried out an optimal dose setting, which accounts for why all the vaccinated mice were able to successfully reject the tumor challenge regardless of how the vaccine was emulsified. In the suboptimal dose tumor challenge experiment (Fig. 7), half of the mice vaccinated with the vortex, syringe-extrusion and homogenizer were still able to successfully reject the tumor. However, in the other half that did develop tumors, it was shown that the mice vaccinated with the emulsion prepared using the vortex technique had the largest tumors and the homogenizer group had the smallest tumors. In the clinical trial settings where the optimal dose might be unknown and where the success of the trial hinges on inducing the maximum immune responses, investigators might want to consider using the syringe-extrusion method or the homogenizer method instead of the vortex to prepare emulsions. Despite the fact that the syringe-extrusion method is more tedious, clinical vaccines are not usually produced for large numbers of patients daily and it would not be that difficult to push the emulsion through a small bore 22-gauge connector as long as it is reinforced with a steel bar. The high-speed homogenizer is the most rapid and convenient method amongst the three techniques compared for preparing W/O emulsions. The model of homogenizer that we have used in our experiment is unsuitable for clinical studies because the homogenizer tip cannot be removed for sterilization but it is the ideal method for investigators carrying out animal studies where large batches of emulsion may need to be made. Omni international manufactures a homogenization system, Omni Tip™ Tissue Homogenizing Kit that features a plastic, sterilizable tip (TH115-PCRD) with a sealed 15 ml tube that could be amenable to clinical use, although this homogenizer system has not been used in our comparison studies.

The depot mechanism by which W/O emulsions exert their adjuvant effect is dependent on the ability of the IFA to form aggregates that can be transported into the lymphoid organs where they will form a stable depot that slowly releases antigen over time[20,21]. This allows the antigen to be processed and presented in the optimal immunological milieu with professional antigen-presenting cells that express co-stimulatory molecules and produce cytokines. Small depots of the emulsions were observed in the draining lymph nodes in the mice that were immunized using syringe-extrusion, vortex and homogenizer (results not shown). All three methods produced emulsions that were able to form aggregates that could be transported into the draining lymph nodes and stably release antigens over time in the environment optimum for the induction of immune response[18] and all three methods can be used for the preparation of peptide-based vaccine emulsions. We have found no differences in the ability of the peptide-based vaccine emulsions to protect against a tumor challenge at an optimal vaccine dose but at suboptimal vaccine dose, the vortex technique was the least able to control tumor growth. The vortex technique induced statistically significantly less IFNγ-secreting cells compared to the syringe technique and had higher variability in the percentage specific lysis in the *in vivo *cytotoxicity assay. This leads us to advise investigators to use the syringe-extrusion technique or homogenizer technique for emulsifying peptide vaccines in clinical trials.

## Abbreviations

IFA: Incomplete Freund's Adjuvant

W/O: Water-in-oil

PBS: Phosphate buffered saline

## Competing interests

The author(s) declare that they have no competing interests.

## Authors' contributions

YTK was responsible for the design of the experiments, the acquisition, analysis and interpretation of the data, as well as the drafting of the manuscript. SAH was involved in the technical aspects of the emulsion preparation and the statistical analysis of the data. JSW participated for intellectual content and brought in the clinical perspectives and expertise. WMK acquired funding, conceptualized the project, and participated in the interpretation of the data and the revisions of the manuscript for final publication. All authors read and approved the final manuscript.
